# 1719. *In Vitro* Activity of Ceftazidime-avibactam and Comparator Agents against Enterobacterales and *Pseudomonas aeruginosa* Collected from Patients with Bloodstream Infections as Part of the ATLAS Global Surveillance Program, 2017-2020

**DOI:** 10.1093/ofid/ofac492.1349

**Published:** 2022-12-15

**Authors:** Mark Estabrook, Gregory Stone, Daniel F Sahm

**Affiliations:** IHMA, Schaumburg, Illinois; Pfizer, Inc., Groton, Connecticut; IHMA, Schaumburg, Illinois

## Abstract

**Background:**

Avibactam (AVI) is a β-lactamase inhibitor with potent inhibitory activity against Class A, Class C, and some Class D serine β-lactamases. The use of ceftazidime (CAZ) with AVI is approved for several indications. This study evaluated the *in vitro* activity of CAZ-AVI and comparators against Enterobacterales (*Eba*) and *Pseudomonas aeruginosa* (*Pae*) isolated from the blood of infected patients as part of the ATLAS surveillance program in 2017-2020.

**Methods:**

A total of 67326 *Eba* and 23051 *Pae* non-duplicate clinically significant isolates, including 14216 *Eba* and 3002 *Pae* isolated from bloodstream infections, were collected in 56 countries in Europe, Latin America, Asia/Pacific (excluding mainland China), and the Middle East/Africa region. Susceptibility testing was performed by CLSI broth microdilution. Meropenem-nonsusceptible (MEM-NS) *Eba* and *Pae* isolates were screened for the presence of β-lactamase genes. Only 25% of MEM-NS *Pae* collected in 2020 were screened.

**Results:**

Of all isolates collected, 97.3% *Eba* and 90.4% *Pae* were susceptible to CAZ-AVI (MIC_90_ values of 0.5 and 8 µg/ml, respectively), more than any comparator tested (table). This was true of blood isolates as well, with 96.9% (*Eba)* and 90.4% (*Pae)* isolates susceptible to CAZ-AVI. CAZ-AVI was active against 89.9% of *Eba* collected from blood that were CAZ-nonsusceptible (NS), more than the comparators tested. While this was not true of CAZ-NS *Pae* blood isolates (52.9% susceptible to CAZ-AVI), the next most active comparator, amikacin, was active against only 3.9 percentage points more isolates. More MEM-NS isolates that screened negative for MBLs were susceptible to CAZ-AVI than any comparator tested (97.8% of *Eba* and 80.1% of *Pae*).

**Figure:**
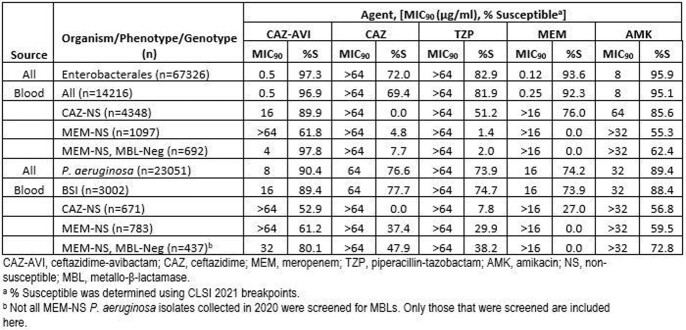

**Conclusion:**

CAZ-AVI provides a valuable therapeutic option for treating bloodstream infections caused by *Eba* and *Pae*, except those carrying MBLs.

**Disclosures:**

**All Authors**: No reported disclosures.

